# 

**Published:** 1881-04

**Authors:** 


WHOLE NUMBER,
CCCCXXXVI.

**April, 1881.**

NUMBER 4,
VOL. XLII.

THE

### CHICAGO MEDICAL Journal and Examiner

**EDITORS:**

N. S. DAVIS, M.D., LL.D. JAS. NEVINS HYDE, A.M., M.D.

DANIEL R. BROWER, M.D.

CHICAGO:

*PUBLISHED BY THE CHICAGO MEDICAL PRESS ASSOCIATION,*

188 CLARK STREET.

☞ **Read the Notice of this Journal among Advertisements.**

